# The use of oximetry and a questionnaire in primary care enables exclusion of a subsequent obstructive sleep apnea diagnosis

**DOI:** 10.1007/s11325-019-01834-2

**Published:** 2019-04-06

**Authors:** Timon M. Fabius, Jeffrey R. Benistant, Rick G. Pleijhuis, Job van der Palen, Michiel M. M. Eijsvogel

**Affiliations:** 1 Department of Pulmonology, Medisch Spectrum Twente, Onderzoeksbureau Longgeneeskunde, P.O. Box 50000, 7500KA Enschede, The Netherlands; 2 Sleep Center, Medisch Spectrum Twente, Enschede, The Netherlands; 3 Department of Research Methodology, Measurement and Data Analysis, Faculty of Behavioural, Management and Social Sciences, University of Twente, Enschede, The Netherlands; 4 Department of Internal Medicine, Medisch Spectrum Twente, Enschede, The Netherlands; 5 Medical School Twente, Medisch Spectrum Twente, Enschede, The Netherlands

**Keywords:** Screening, Primary care, Obstructive sleep apnea, Oximetry, Questionnaire

## Abstract

**Purpose:**

The study aims to prospectively validate the prognostic value of oximetry alone or combined in a two-step strategy with a questionnaire for the exclusion of obstructive sleep apnea (OSA) in primary care.

**Methods:**

A total of 140 subjects with suspected OSA were included from 54 participating primary care practices. All subjects completed the Philips questionnaire and underwent one night of oximetry prior to referral to a sleep center. The prognostic value of two strategies was evaluated against the diagnosis of the sleep center as the gold standard: (1) assume OSA and subsequently refer to a sleep center if the oxygen desaturation index (ODI) is ≥ 5 and (2) assume OSA and refer to a sleep center if the Philips questionnaire score is ≥ 55% (regardless of the ODI) or if the Philips questionnaire score is < 55% and the ODI is ≥ 5.

**Results:**

OSA was diagnosed in the sleep centers in 100 (71%) of the included subjects. Using ODI ≥ 5 alone resulted in a sensitivity of 99.0%, a specificity of 50.0%, a negative predictive value of 95.2%, and a positive predictive value 83.2%. Using the two-step strategy, oximetry would be performed on 39% of the subjects. This strategy resulted in a sensitivity of 100%, a specificity of 35.0%, a negative predictive value of 100%, and a positive predictive value of 79.4%.

**Conclusions:**

In a Dutch primary care population with a clinical suspicion of OSA and low frequency of cardiovascular comorbidities, the use of oximetry alone or combined in a two-step strategy with a questionnaire enables exclusion of a sleep center diagnosis of OSA.

**Electronic supplementary material:**

The online version of this article (10.1007/s11325-019-01834-2) contains supplementary material, which is available to authorized users.

## Introduction

Obstructive sleep apnea (OSA) is a common sleep disorder that causes patients to stop breathing during sleep. Over the past decades, the hazardous effects of OSA on personal health [1] and society [2, 3] have become more and more clear. Still, it is estimated that more than half of all patients suffering from OSA are undiagnosed and therefore untreated [4].

Diagnosing OSA and setting up an appropriate treatment requires specialized care that is generally only available in sleep clinics. In the Netherlands, the number of referrals to sleep clinics for OSA approached 100.000 in 2017 and has increased rapidly over the past few years [5]. Of all patients referred by their general practitioner, up to one third (30%) eventually does not have OSA upon final poly(somno)graphy [5]. This may be explained by the heterogeneous and often non-specific symptoms of OSA, making it difficult to distinguish OSA from other diagnoses.

Simply increasing the number of referrals to sleep clinics would result in more treated OSA patients, but also in an unacceptable rise in costs due to an associated increase in expensive poly(somno)graphies performed in patients without OSA. In addition, the capacity of sleep clinics is limited, resulting in (extended) waiting lists when the number of referred patients grows. To maximize the number of treated OSA patients without increasing costs or waiting lists, we propose to focus on optimization of the selection process regarding which patients should (not) be referred.

In an attempt to triage referral based on the pre-test probability of OSA, several screening strategies have been developed. Examples include questionnaires (e.g., STOP-BANG [6, 7], Berlin [8], Epworth Sleepiness Scale [9]) or a two-step screening strategy (e.g., the Philips questionnaire combined with nasal flow recording [10]). However, although some are suitable for screening in low-prevalence populations, none of these have shown acceptable sensitivity to safely rule out OSA when used in a high-prevalence referral population. In 2016, Kunisaki et al. reported on a prospective observational study in which overnight oximetry was applied to detect OSA in 234 veterans referred for sleep testing [11]. Based on a positive predictive value of 92 to 100%, the authors concluded that overnight oximetry could significantly reduce the number of patients requiring referral for polysomnography.

In this study, we hypothesized that overnight oximetry alone, or combined with a previously published questionnaire in a two-step strategy, could be used to safely rule out OSA in patients visiting their general practitioner with potentially OSA-related complaints, thereby reducing the number of patients requiring referral for sleep testing. We prospectively validated this hypothesis in a high-prevalence population of patients with suspected OSA in the general practice setting.

## Methods

### Participants

This was a prospective observational validation study in 54 general practitioner practices located in the catchment area of the sleep centers of two large teaching hospitals. All adult (≥ 18 years) subjects referred to one of the sleep centers by a participating general practitioner due to suspected OSA were eligible for inclusion. Patients were approached and included by their general practitioner. Subjects who were unable to undergo oximetry or had missing data (i.e., missing questionnaire, oximetry, or sleep center diagnosis) were excluded from the analysis.

All participating subjects provided written informed consent. The study protocol was approved by the Medical Ethical Committee Twente (Enschede, the Netherlands) and registered at the Netherlands Trial Register (www.trialregister.nl, ID: NTR5786).

### Measurements

Included subjects completed an online version of the Philips questionnaire [10] and underwent overnight oximetry using the Nonin WristOx_2_^™^ model 3150 wrist-worn pulse oximeter (Nonin Medical, Inc., Plymouth, MN, USA). The questionnaires were completed without the presence or further explanation of a health care professional. Of the patient characteristics body weight and height were patient reported. Neck circumference was measured at the general practitioner’s practice. For the oximetry, subjects were instructed to start the measurement when they went to bed. The oxygen desaturation index (ODI; number of saturation drops (≥ 3%) divided by recording time) was automatically obtained from the oximetry data using a custom-build script in Matlab (The Mathworks, Inc., Natick, MA, USA).

All measurements were performed before OSA was diagnosed or excluded in the sleep center. The diagnosis or exclusion of OSA in the sleep center was based on regular care (i.e., symptoms, medical history, physical examination, and poly(somno)graphy). Similar to those of the American Academy of Sleep Medicine, the Dutch guideline for diagnosis and treatment of OSA in adults recommends a diagnosis of OSA if poly(somno)graphy results in an apnea-hypopnea index (AHI) ≥ 15 or an AHI between 5 and 15 combined with specific symptoms or comorbidities [12, 13]. The poly(somno)graphy data were analyzed according to the American Academy of Sleep Medicine guidelines [14]. Desaturation were defined as a ≥ 3% decrease from pre-event baseline. In polygraphy, hypopneas had to be associated with a desaturation. In polysomnography, hypopneas had to be associated with either a desaturation or an arousal. In case of OSA associated symptoms but a negative polygraphy (i.e., AHI < 5), a polysomnography was performed (as per AASM and Dutch guidelines). The health care professionals of the sleep centers were unaware of the results of the questionnaire and overnight oximetry.

### Statistical analysis

The primary aim of this study was to validate a predefined strategy to rule out OSA using oximetry alone or combined with the Philips questionnaire in a two-step strategy. For oximetry, an ODI < 5 has high resemblance with an AHI < 5 when measured simultaneously [15]. Therefore, an ODI < 5 was chosen as cutoff for the oximetry. The Philips questionnaire was originally developed in a population of healthy blue- and white-collar workers and results in a score ranging from 0 to 100%. A score below 35% indicated a low risk on OSA, a score between 35 and 55% an intermediate risk, and a score of 55% and above a high risk [10]. The development and validation (along with a full text English version of the questionnaire) are published elsewhere [10]. In the current study, the questionnaire was combined in a two-step strategy by ruling out OSA in those patients with an ODI < 5 and a score on the Philips questionnaire below 55% (i.e., those with an ODI ≥ 5 or a high risk on OSA according to the Philips questionnaire should be referred to a sleep center).

The results of the oximetry alone and the two-step strategy were compared with the diagnosis from the sleep centers using cross-tabulation. Subsequently, sensitivity, specificity, negative predictive value, positive predictive value, and area under the receiver operating characteristics curve (AUC) were calculated. Data were analyzed using SPSS, version 24 (SPSS Inc., Chicago, IL, USA).

A secondary exploratory analysis was performed to identify optimal cutoffs for the questionnaire and oximetry for the exclusion of OSA.

### Sample size

Given the burden of untreated OSA, a high sensitivity is desirable. For this study, a sensitivity of 97% with a precision of 10% and a lower boundary of the 95% confidence interval (95% CI) of 90% was deemed acceptable. To achieve this, 68 subjects with subsequent sleep center diagnosis of OSA should be included. The prevalence of OSA in patients referred to the participating sleep centers is approximately 70%. It was expected that the general practitioners might be keener on referring patients with suspected OSA due to study participation, resulting in a lower prevalence of OSA in those referred. The prevalence in the study population was therefore estimated at 50%, resulting in 136 subjects needed with complete data. With an estimated dropout of 10%, it was expected that a total of 150 subjects needed to be included.

## Results

**Fig. 1:**
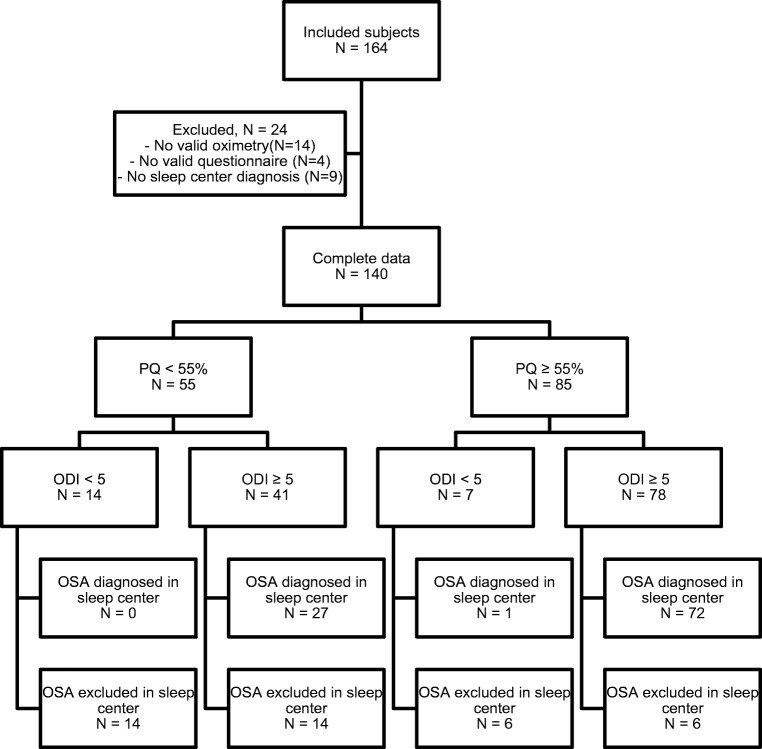
Flowchart of the included subjects

Of 164 included subjects, 140 had complete data (see flowchart; Fig. 1). Of these, 119 (85%) had an ODI ≥ 5 and 85 (61%) a Philips questionnaire score ≥ 55%. OSA was diagnosed in the sleep centers in 100 subjects (71%). Characteristics of the analyzed subjects are provided in Table 1.

**Table 1:** Characteristics of the included subjects

	Total (*N* = 140)	OSA diagnosed in sleep center (*N* = 100)	OSA excluded in sleep center (*N* = 40)	*P* value
General characteristics				
Age, years (SD)	49.3 (13.7)	52.4 (12.6)	41.4 (13.1)	< 0.001
Males, *N* (%)	101 (72.1)	78 (78.0)	23 (57.5)	0.02
Weight, kg (SD)	96.3 (18.0)	99.4 (18.2)	88.6 (15.0)	0.001
Body mass index, kg/m^2^ (IQR)	29.4 (25.7–33.3)	30.7 (26.4–34.4)	26.7 (24.7–29.4)	< 0.001
Heart failure, *N* (%)	1 (0.7)	1 (1.0)	0 (0.0)	1.00
Cardiac ischemia, *N* (%)	7 (5.0)	6 (6.0)	1 (2.5)	0.67
Atrial fibrillation, *N* (%)	9 (6.4)	9 (9.0)	0 (0.0)	0.06
Hypertension, *N* (%)	35 (25.0)	33 (33.0)	2 (5.0)	0.001
Hypercholesterolemia, *N* (%)	12 (8.6)	10 (10.0)	2 (5.0)	0.509
Diabetes mellitus, *N* (%)	12 (8.6)	11 (11.0)	1 (2.5)	0.18
Malignancy, *N* (%)	6 (4.3)	6 (6.0)	0 (0.0)	0.18
Hypothyroidism, *N* (%)	9 (6.4)	4 (4.0)	5 (12.5)	0.12
Stroke, *N* (%)	5 (3.6)	4 (4.0)	1 (2.5)	1.00
COPD, *N* (%)	3 (2.1)	2 (2.0)	1 (2.5)	1.00
Measured by the general practitioner				
Neck circumference, cm (SD)	40.6 (3.9)	41.5 (3.6)	38.4 (3.6)	< 0.001
Oxygen desaturation index (IQR)	11.9 (6.1–22.1)	16.2 (10.6–25.4)	5.1 (3.1–7.0)	< 0.001
Oxygen desaturation index ≥ 5, *N* (%)	119 (85.0)	99 (99.0)	20 (50.0)	< 0.001
Philips questionnaire, % (IQR)	70.1 (46.6–93.1)	89.2 (51.8–94.8)	46.6 (28.0–65.8)	< 0.001
Philips questionnaire ≥ 55%, *N* (%)	85 (60.7)	73 (73.0)	12 (30.0)	< 0.001
Measured at the sleep center				
Epworth Sleepiness Scale (SD, *N* = 125)	7.3 (4.5)	7.6 (4.7)	6.6 (4.1)	0.30
Apnea-hypopnea index (IQR)	13.6 (6.3–23.8)	18.2 (12.9–31.8)	3.3 (2.5–4.8)	< 0.001
Apnea index (IQR)	1.0 (0.4–5.5)	2.8 (0.5–9.2)	0.3 (0.0–0.7)	< 0.001
Hypopnea index (IQR)	10.1 (4.7–16.6)	13.7 (8.6–18.7)	2.8 (1.5–4.3)	< 0.001
Apnea-hypopnea index 5–15, *N* (%)	47 (33.6)	38 (38.0)	9 (22.5)	0.079
Apnea-hypopnea index 15–30, *N* (%)	35 (25.0)	35 (35.0)	0 (0.0)	< 0.001
Apnea-hypopnea index ≥ 30, *N* (%)	27 (19.3)	27 (27.0)	0 (0.0)	< 0.001

Data are presented as mean with standard deviation (SD), median with interquartile range (IQR), or number with corresponding percentage

Using an ODI < 5 alone ruled out OSA in 21 (15%) subjects of which one did have OSA diagnosed in the sleep center. Cross-tabulation of the use of ODI alone against the sleep center diagnosis is provided in Table 2. This strategy resulted in a sensitivity of 99.0% (95% CI 94.5–100.0%), a specificity of 50.0% (95% CI 33.8–66.2%), a negative predictive value of 95.2% (95% CI 76.2–99.9%), a positive predictive value of 83.2% (95% CI 75.2–89.4%), a positive likelihood ratio of 1.98 (95% CI 1.45–2.70), a negative likelihood ratio of 0.02 (95% CI 0.00–0.14), and a corresponding AUC of 0.75 (95% CI 0.64–0.85). The subject who was diagnosed with OSA in the sleep center while the screening oximetry resulted in an ODI < 5 had a score of 98% on the Philips questionnaire, an ODI of 3/h, and a respiratory event index in the sleep center of 6.4/h as measured by polygraphy.

**Table 2:** Cross-tabulation of the use of oximetry alone versus the diagnosis of the sleep centers

	OSA diagnosed	OSA excluded	Total
ODI ≥ 5	99	20	119
ODI < 5	1	20	21
Total	100	40	140

ODI oxygen desaturation index

The two-step strategy to refer to the sleep center if the Phillips questionnaire is ≥ 55% or the ODI is ≥ 5 (i.e., OSA is only excluded if the Philips questionnaire is < 55% and ODI < 5) ruled out OSA in 14 (10%) subjects of which none had OSA diagnosed in the sleep centers. Cross-tabulation of the two-step strategy against the sleep center diagnosis is provided in Table 3. This strategy resulted in a sensitivity of 100% (95% CI 96.3–100.0), a specificity of 35.0% (95% CI 20.6–51.7%), a negative predictive value of 100% (95% CI 76.8–100.0%), a positive predictive value of 79.4% (95% CI 71.2–86.1%), a positive likelihood ratio of 1.54 (95% CI 1.23–1.93), a negative likelihood ratio of 0.00 (95% CI not applicable), and a corresponding AUC of 0.68 (95% CI 0.57–0.79).

**Table 3:** Cross-tabulation of the two-step strategy alone versus the diagnosis of the sleep centers

	OSA diagnosed	OSA excluded	Total
PQ ≥ 55% or ODI ≥ 5	100	26	126
PQ < 55% and ODI < 5	0	14	14
Total	100	40	140

PQ Philips questionnaire, ODI oxygen desaturation index

The explorative analysis showed that an optimal combination of sensitivity and specificity could be achieved by assuming OSA (and subsequently refer to the sleep center) if one of the following three conditions applied: (1) the Philips questionnaire was ≥ 92%, or (2) the ODI rounded to the nearest integer was ≥ 10, or (3) the rounded ODI was between 5 and 10 and the Philips questionnaire was ≥ 46.5%. Cross-tabulation of these optimized cutoffs versus the final diagnosis of the sleep centers is provided in Table 4. These cutoffs would result in a sensitivity of 99.0% (95% CI 94.6–100.0%), a specificity of 65.0% (95% CI 48.3–79.4%), a negative predictive value of 96.3% (95% CI 81.0–99.9%), a positive predictive value of 87.6% (95% CI 80.1–93.1%), a positive likelihood ratio of 2.83 (95% CI 1.85–4.32), a negative likelihood ratio of 0.02 (95% CI 0.00–0.11), and a corresponding AUC of 0.82 (95% CI 0.73–0.91).

**Table 4:** Cross-tabulation of the optimal (according to the post hoc analysis) two-step strategy versus the diagnosis of the sleep centers

	OSA diagnosed	OSA excluded	Total
Optimal strategy positive	99	14	113
Optimal strategy negative	1	26	27
Total	100	40	140

This strategy was deemed positive (i.e., OSA is assumed likely and a subject should be referred to a sleep center) if (1) the Philips questionnaire was ≥ 92%, or (2) the ODI rounded to the nearest integer was ≥ 10, or (3) the rounded ODI was between 5 and 10 and the Philips questionnaire was ≥ 46.5%

OSA obstructive sleep apnea, ODI oxygen desaturation index

## Discussion

The primary aim of this study was to validate the use of oximetry alone or combined with a questionnaire to exclude OSA in primary care. The results show that both pre-defined strategies have a high sensitivity (slightly higher for the two-step strategy) and moderate specificity (slightly higher for oximetry alone). The strength of oximetry alone is the higher specificity, which would significantly decrease the number of subjects referred for further workup. However, this strategy seems to erroneously exclude OSA in approximately 1% of the subjects with subsequently diagnosed OSA when referred to a sleep center, whereas no OSA case was missed using the two-step strategy. Furthermore, if the two-step strategy is applied, oximetry would only be needed in 39% of the patients. On the other hand, the two-step strategy excludes OSA in fewer subjects (10 versus 15%). Moreover, the respiratory event index resulting from the sleep center analysis in the one false-negative subject in the oximetry-only strategy was only slightly higher than the cutoff of five events per hour. This may have been caused by the known night-to-night variation in OSA severity [16]. Both strategies seem to have their strengths and the exact costs will strongly depend on the local costs of an oximetry reading and the local health care system. An extensive cost-effectiveness analysis elaborating on the abovementioned screening strategies was recently published elsewhere [17].

The exploratory analysis resulted in cutoffs that can substantially increase the specificity of the two-step strategy with only a limited decrease in sensitivity. This would add substantially to the clinical applicability of the strategy. However, it would require oximetry recordings in all subjects, whereas the predefined cutoffs would only require oximetry in those with a low to moderate score on the Philips questionnaire. Again, whether the added costs of the oximetry reading will outweigh the saved costs of the prevented sleep center referrals strongly depends on the local health care system. More importantly, the optimized cutoffs should be validated prospectively before they can be used in clinical practice.

The findings of this study confirm that a referral to a sleep center for the diagnosis of OSA can be omitted in primary care using oximetry alone or a two-step strategy combining oximetry and a questionnaire. A recent study in Spain showed that the workup and management of OSA in primary care was non-inferior to the workup and management of OSA in a specialized sleep center [18]. Similar results were reported earlier from a study in Australia [19]. We aimed to validate the exclusion of OSA in primary care rather than its confirmation and management. Although the mentioned trials suggest that management is also feasible, it is important to note that both studies only included uncomplicated patients and management was performed by trained nurses. In our study, only subjects who were unable to undergo oximetry or refused informed consent were excluded. Furthermore, no strict inclusion criteria were applied. The findings of our study seem therefore applicable to a broader population. It should be noted, however, that our study was not powered to validate the proposed strategies in subpopulations (e.g., subjects with several significant comorbidities).

With the right inclusion and exclusion criteria one might argue that an aberrant oximetry recording alone might be sufficient to start a CPAP trial. However, the use of oximetry alone (rather than a poly(somno)graphy recording) has inherent limitations. Foremost, an oximetry reading provides much less information than a poly(somno)graphy recording. For instance, oximetry will not allow to differentiate between central and obstructive events or if there is a significant positional dependence. Both might enable (or even require) other treatment strategies than plain CPAP. Nevertheless, oximetry alone may be sufficient for a selected (sub) group of patients. The key will lie in the way this group of patients is selected. A large Australian study already investigated the use of full  polysomnography versus polygraphy versus oximetry as the basis for the management of OSA [20]. The results showed that oximetry alone (compared to polysomnography) resulted in shorter CPAP usage and less improvement in reported sleep apnea symptoms. Though subjects with significant comorbidities were excluded, all ranges of the ODI were used for management decisions. One might argue that the combination of a high ODI (e.g., > 30 or 40/h), the absence of significant comorbidities (such as heart failure and neuromuscular conditions), and a high clinical probability of OSA (according to a sleep expert) might enable selection of those patients in whom CPAP can be started without additional testing. If (as was done in our study) the ODI is calculated automatically, we recommend manual affirmation of the automated analysis. In addition, future prospective studies are needed to investigate this interesting hypothesis. Our study has some limitations. First, the clinical diagnosis rather than a test result was used as reference standard. This choice was based on the recent reports indicating a very high prevalence of an increased AHI in the general population of which many do not have any symptoms [21]. Although the debate on whether all asymptomatic individuals with an increased AHI can be left untreated is still ongoing, we feel that the diagnosis of OSA should be based on the combination of AHI with symptoms. This is partly reflected in our results, as OSA was not diagnosed in 10 subjects with an elevated AHI. A second point worthy of note is the number of included subjects. Based on the sample size analysis, 68 subjects in whom OSA was subsequently diagnosed should have been included. However, 100 were included. This was mainly caused by a delay between inclusion (and study measurements) and the time of diagnosis in the sleep centers. Another noteworthy element is that a negative screening result does not exclude the presence of other sleep pathology. If implemented in clinical practice, it should be emphasized that the screening strategies are only capable of excluding OSA and subsequent referral to a sleep center for some other sleep pathology might still be useful. The addition of other (non-OSA) sleep-related questions might help the general practitioner to recognize such cases. As an example, the Athens Insomnia Scale [22] is already incorporated in the Philips questionnaire [10].

Finally, although it was not an exclusion criterion, the prevalence of comorbidities that might significantly influence the results of an oximetry reading such as COPD, heart failure, or neuromuscular disorders was low in the study population. Furthermore, the study population was predominantly male. This may reflect clinical practice but there are significant differences in OSA pathophysiology and presentation between males and females [23]. This may influence the accuracy of diagnostic strategies. We did not observe large differences in accuracy of the three presented strategies between males and females (see supplementary data). However, our study was not powered to prove differences in diagnostic accuracy between males and females. This precludes a firm conclusion on the use of oximetry for OSA screening in females and subjects with specific comorbidities. Future studies should address the accuracy of oximetry (combined with a questionnaire) in specific comorbidities and females.

Summarizing, in a Dutch primary care population with a clinical suspicion of OSA and low frequency of cardiovascular comorbidities, the use of oximetry alone or combined in a two-step strategy with a questionnaire enables exclusion of a sleep center diagnosis of OSA. The two-step strategy with oximetry only in those with a low probability for OSA (based on the Philips questionnaire) results in a higher sensitivity but lower specificity when compared with oximetry alone. The two-step strategy may be more cost-effective as fewer oximetry recordings would be needed.

## Electronic supplementary material

ESM 1 (DOCX 14 kb)

## Abbreviations

OSA: Obstructive sleep apnea
ODI: Oxygen desaturation index
AHI: Apnea-hypopnea index
AUC: Area under the receiver operating characteristics curve
95% CI: 95% confidence interval
SD: Standard deviation
IQR: Interquartile range
