# Supplementary material for: The use of oximetry and a questionnaire in primary care enables exclusion of a subsequent obstructive sleep apnea diagnosis
Source: Sleep Breath. 2019 Apr 6;24(1):151–8. doi: 10.1007/s11325-019-01834-2 (PMC7127990; doi:10.1007/s11325-019-01834-2)
Supplement: Supplementary file 1 — (DOCX 14 kb) [file 11325_2019_1834_MOESM1_ESM.docx]

Supplementary data to the article “The use of oximetry and a questionnaire in primary care enables safe exclusion of a subsequent obstructive sleep apnea diagnosis”.

Authors: TM Fabius, JR Benistant, RG Pleijhuis, J van der Palen, MMM Eijsvogel.

Table S1 – Diagnostic accuracy of the three tested strategies for the sleep center diagnosis of OSA in males and females. The explorative analysis was deemed positive if 1) the Philips questionnaire was ≥ 92%, or 2) the ODI rounded to the nearest integer was ≥ 10, or 3) the rounded ODI was between 5 and 10 and the Philips questionnaire was ≥ 46.5%.

|  | ODI ≥ 5 | | ODI ≥ 5 or PQ ≥ 55% | | Explorative analysis | |
| --- | --- | --- | --- | --- | --- | --- |
|  | Sens (95%CI) | Spec ((95%CI) | Sens (95%CI) | Spec (95%CI) | Sens (95%CI) | Spec (95%CI) |
| Total (N=140) | 99,0 (94,6 - 100,0) | 50,0 (33,8 - 66,2) | 100,0 (96,4 - 100,0) | 35,0 (20,6 - 51,7) | 99,0 (94,6 - 100,0) | 65,0 (48,3 - 79,4) |
| Males (N=101) | 98,7 (93,1 - 100,0) | 52,2 (30,6 - 73,2) | 100,0 (95,4 - 100,0) | 30,4 (13,2 - 52,9) | 98,7 (93,1 - 100,0) | 69,6 (47,1 - 86,8) |
| Females (N=39) | 100,0 (84,6 - 100,0) | 47,1 (23,0 - 72,2) | 100,0 (84,6 - 100,0) | 41,2 (18,4 - 67,1) | 100,0 (84,6 - 100,0) | 58,8 (32,9 - 81,6) |

OSA: Obstructive Sleep Apnea; ODI: Oxygen Desaturation Index; PQ: Philips Questionnaire;
